# A High-Sugar Diet Consumption, Metabolism and Health Impacts with a Focus on the Development of Substance Use Disorder: A Narrative Review

**DOI:** 10.3390/nu14142940

**Published:** 2022-07-18

**Authors:** Kacper Witek, Karolina Wydra, Małgorzata Filip

**Affiliations:** Department of Drug Addiction Pharmacology, Maj Institute of Pharmacology Polish Academy of Sciences, Smętna Street 12, 31-343 Kraków, Poland; witek@if-pan.krakow.pl (K.W.); wydra@if-pan.krakow.pl (K.W.)

**Keywords:** sugar, carbohydrates, sucrose, glucose, fructose, high-sugar diet, predictors of SUD, substance use disorder, vulnerability to SUD

## Abstract

Carbohydrates are important macronutrients in human and rodent diet patterns that play a key role in crucial metabolic pathways and provide the necessary energy for proper body functioning. Sugar homeostasis and intake require complex hormonal and nervous control to proper body energy balance. Added sugar in processed food results in metabolic, cardiovascular, and nervous disorders. Epidemiological reports have shown enhanced consumption of sweet products in children and adults, especially in reproductive age and in pregnant women, which can lead to the susceptibility of offspring’s health to diseases in early life or in adulthood and proneness to mental disorders. In this review, we discuss the impacts of high-sugar diet (HSD) or sugar intake during the perinatal and/or postnatal periods on neural and behavioural disturbances as well as on the development of substance use disorder (SUD). Since several emotional behavioural disturbances are recognized as predictors of SUD, we also present how HSD enhances impulsive behaviour, stress, anxiety and depression. Apart from the influence of HSD on these mood disturbances, added sugar can render food addiction. Both food and addictive substances change the sensitivity of the brain rewarding neurotransmission signalling. The results of the collected studies could be important in assessing sugar intake, especially via maternal dietary patterns, from the clinical perspective of SUD prevention or pre-existing emotional disorders. Methodology: This narrative review focuses on the roles of a high-sugar diet (HSD) and added sugar in foods and on the impacts of glucose and fructose on the development of substance use disorder (SUD) and on the behavioural predictors of drugs abuse. The literature was reviewed by two authors independently according to the topic of the review. We searched the PubMed and Scopus databases and Multidisciplinary Digital Publishing Institute open access scientific journals using the following keyword search strategy depending on the theme of the chapter: “high-sugar diet” OR “high-carbohydrate diet” OR “sugar” OR “glucose” OR “fructose” OR “added sugar” AND keywords. We excluded inaccessible or pay-walled articles, abstracts, conference papers, editorials, letters, commentary, and short notes. Reviews, experimental studies, and epidemiological data, published since 1990s, were searched and collected depending on the chapter structure. After the search, all duplicates are thrown out and full texts were read, and findings were rescreened. After the selection process, appropriate papers were included to present in this review.

## 1. Introduction

Carbohydrates (=saccharides) are groups of chemical compounds that include sugars, starch, and cellulose [1]. Saccharides are divided into four chemical subgroups according to the degree of polymerization by indication of the length of the carbohydrate chain: monosaccharides (with only 1 sugar unit), disaccharides (with 2 sugar units), oligosaccharides (containing 3–9 sugar units), and polysaccharides (with ≥10 sugar units) (Table 1). The representatives of the monosaccharides are glucose, galactose, and fructose; examples of disaccharides include sucrose (glucose + fructose), maltose (2 glucose units) and lactose (glucose + galactose); and the polysaccharides are starches. Carbohydrates are crucial to nutrition and perform crucial roles in organisms. Monosaccharides are a source of energy for cells, and some biomolecules fulfil numerous roles in biosynthesis and build other subgroups of saccharides. Mono- and disaccharides act as sweeteners in many foods, as well as natural preservatives. Fructose is the sweetest of all food carbohydrates. Polysaccharides are used for energy storage and as structural components [2,3]. Processed, refined sugars (called added sugars), such as sucrose or high-fructose corn syrup (HFCS), provide little nutritional value to food and consist of empty calories. High values of added sugars appear in sugar-sweetened beverages (SSBs). Excess sugar bingeing is dangerous for the human body, contributing to the epidemic of obesity and overweight [4,5].

### 1.1. Daily Limits for Carbohydrate Intake

Dietary carbohydrates are crucial to nutrition and are found in a wide variety of natural and processed foods. The Institute of Medicine (IOM) established a limit for carbohydrates of 130 g per day for adults and children aged ≥1 year old. The IOM and the Dietary Guidelines for Americans recommend that carbohydrates should provide 45–65% of human daily caloric intake for all ages and genders [6,7]. Moreover, according to the Food and Drug Administration, the daily value for carbohydrates is 300 g per day in humans consuming a 2000-calorie diet [8]. In turn, the daily sugar limit for laboratory animals depends on the species, stage of development, strain, and individual preferences. In most cases, standard rodent diets are based on the safety margins reported by laboratories. To date, a specific need for carbohydrates has not been established, but rats are better at eating a diet rich in sugar, such as glucose, or its precursors, such as starch. Diets high in fat or sugar have contributed to the promotion of obesity and overweight. It should be mentioned that fructose and sucrose (as a source of fructose) can lead to anomalies compared with glucose [9,10]. The control laboratory rodent diet should contain between 55% and 65% of its energy requirement from carbohydrates, with additional fibre, at the required temperature and humidity conditions [10,11,12,13]. 

### 1.2. Added Sugar Consumption

In 2008 and 2012, adults in the United States consumed more than 28 kg of added sugar. The average sugar consumption in 2008 was 76.7 g (19 teaspoons), and in 2012, it was 77 g per day [14,15,16]. The average daily intake of added sugars to food in 2017–2018 was 17 teaspoons per day for children and adults in the USA. In particular, men consume 20% more teaspoons of added sugar than women [17]. In 2011–2014, American youth and adults consumed an average of 143 and 145 calories from SSB, respectively [18,19]. Currently, the World Health Organization (WHO) guideline recommends reducing free sugar (including monosaccharides and disaccharides) intake to less than 10% of total daily energy intake in adults and children, whereas the Institute of Medicine recommends that added sugar make up less than 25% of total calories [20,21]. Clinical trials and epidemiologic studies have shown that individuals who consume greater amounts of added sugar, especially sugar-sweetened beverages, tend to gain more weight and have a higher risk of obesity, type 2 diabetes mellitus, dyslipidaemia, hypertension, and cardiovascular disease [22].

Since 1990, the most important change in the eating patterns of United Kingdom consumers has been a reduction in high-fat milk consumption, especially among pre-schoolers, so children and adolescents have switched to sodas, fruit drinks, juices, and sweetened dairy products [23]. Additionally, in the UK, the total sales volume of SSB decreased, whereas volume sales of low- and zero-sugar drinks and water increased between 2015 and 2018 [24]. The consumption of fructose is high in Western diets, mainly due to the consumption of refined or processed sugars, such as HFCS [25]. In the United States, HFCS is found in nearly all processed foods that contain sweeteners. HFCS—due to its low cost of production—replaced sucrose as a sweetener [26].

Currently, little clinical research data about fructose and HFCS consumption are available [27]. Unfortunately, no clear dietary recommendations on fructose or HFCS consumption limits are available. Additional research is needed to determine the adverse effects of consuming added fructose-containing sugars. Several previous data have indicated that total daily caloric intake in the form of added sugars increased over the study period (1970–1999) [28], including fructose availability in grams per day [29] and total fructose intake [30,31]. Further research is needed to understand the impact of increased intake of fructose on human health. More than 10% of Americans daily calories come from fructose consumption [28]. When too much sugar or too many carbohydrates are eaten, all this energy must basically be stored inside the fat cells or fat droplets, and this fat can accumulate in internal organs, especially in the liver but also peripherally—subcutaneously. Many more calories from fructose are converted into abdominal fat, whereas less fat from glucose accumulates in the liver. HSDs, sucrose, and thus glucose and fructose cause dysregulation of lipid and carbohydrate metabolism in the body. Added sugar bingeing promotes an increased energy balance, weight gain, fat storage and consequently overweight and/or obesity [32,33,34,35]. An increase in high-sugar products and added sugar consumption has been observed since the 1980s (more visible in the USA) and suggests that sugar, not including fat, is the major factor contributing to and driving the current obesity epidemic and diabetes [36,37]. 

According to a WHO report, in 2016, more than 1.9 billion adults (18 years and older) were overweight, and over 650 million of them, mainly women were obese [38]. Glucose metabolism disturbances can occur in neurological diseases and have been reported in both clinical and preclinical studies. Alzheimer’s [39,40,41] and Parkinson’s diseases [42] lead to a decrease in glucose levels or degeneration of neurons, also resulting from a diet rich in added sugars [43]. In contrast, in a rat infarction model, an increase in glucose levels in the ischaemic area was observed [44]. Interestingly, in addition to changes in sugar metabolism in the nervous system, sugar itself could exert a protective effect by increasing glucose uptake by nerve cells and restoring adequate metabolic homeostasis [45,46]. Excessive consumption of fructose can lead to inflammation in the nervous system, microglial overactivation, and oxidative stress. Preclinical research has also suggested cognitive and memory impairment in rodents fed high concentrations of fructose [47] or sugar [48,49]. Sugar consumption impacts brain diseases or disorders, such as anxiety, depression, neurogenesis, fear, and changes in molecular and neurochemical expression [50]. 

The basic animal research model for examining the effects of sugar intake is to provide them with sweet food. Such modified diet consists predominantly of food enriched with sweetened (with sucrose, glucose, or fructose) beverages or HFCS or, to a lesser extent, food enriched only with a high content of sucrose, fructose, glucose, or other sugars in feed [20]. Animal models of excess sugar intake in the diet produce obesity, and this effect is mainly under investigation [51,52]. In animal modelling studies (but not in human research), it is possible to measure the amount of sweet food consumption and energy balance due to strictly restrictive limits on types of food, liquids, or number of calories.

### 1.3. Carbohydrate ADME Processes

The fate of carbohydrates in an organism can be described by the absorption, distribution, metabolism, and excretion (ADME) process. In mammals, dietary di-, oligo- and polysaccharides are digested in the mouth, stomach, and intestine and are broken down into monosaccharides by special (salivary amylase, stomach acid, and specific carbohydrases—glycoside hydrolases, respectively) enzymes, and finally, the small intestine absorbs them into the bloodstream. Cellulose dietary fibres are indigestible carbohydrates fermented in the large intestine using the presence of bacteria [53]. Monosaccharides are transported through the hepatic portal vein to the liver. Fructose and galactose are phosphorylated to glucose by fructo- and galactokinase. Glucose is metabolized in all body cells by glycolysis [54]. This metabolic pathway converts 1 glucose molecule into 2 pyruvic acid molecules—the substrate for the citric acid cycle (CAC). Glycolysis also releases 2 high-energy adenosine triphosphate (ATP) molecules and 2 molecules of reduced nicotinamide adenine dinucleotide. The complete breakdown of one glucose molecule by aerobic respiration (glycolysis + CAC) forms approximately 30–33 molecules of ATP [55]. Glucose serves as a substrate of cellular respiration or is stored as glycogen in glycogenesis [14]. 

Monosaccharides, including glucose and fructose, enter mammalian cells via two different types of membrane-associated carrier proteins: sodium-glucose linked transporters (SGLTs) and facilitated diffusion glucose transporters (GLUTs) [56,57,58]. SGLT and GLUT expression occurs in tissues (e.g., in the intestine, kidney, and liver) where energy requirements or sugar biotransformation is necessary for other metabolic pathways (Table 2). Additionally, adult brain neurons are in high energy demand; therefore, transporter presence is found in the brain [59] (Table 2). Neurons consume approximately 20% of glucose-derived energy, making them the main consumer of glucose. Glucose enters the mammalian brain from the blood across the blood–brain barrier (BBB), which galactose and fructose are believed to cross. The large blood-brain concentration gradient drives the facilitating transport of glucose across endothelial membranes via GLUT1 glucose transporters into the extracellular fluid. GLUT1 further mediates glucose uptake from the extracellular fluid into astrocytes, oligodendroglia, and microglia, whereas GLUT3, which has a much higher transport rate than GLUT1, facilitates neuronal glucose uptake [45]. The expression of GLUT1 is much higher than that of GLUT3 in the developing and prenatal brain. In neurons and astrocytes, glucose is the source of pyruvate to fuel the citric acid cycle for the production of ATP. The SGLTs that mediate secondary active transport are likely not active in healthy conditions but rather in pathologic situations [60]. In contrast to glucose (80–120 mg/dL) [61,62], circulating levels of fructose in blood plasma are extremely low (<0.050 mM) [63,64]. The process of transporting fructose from the blood to the brain is unclear, and the data collected remain inconclusive. Among the expression of active fructose transporters (Table 2), such as SGLT4, GLUT5, and GLUT8 [4,64,65,66], only GLUT5 has been identified in the human and rat BBB [67,68].

The most important organ regulating carbohydrate metabolism in mammals is the liver. Here, the control of energy homeostasis is mainly reduced to regulation of the level of glucose in the blood. Glucose delivered to hepatocytes leads to de novo lipogenesis (DNL), the synthesis of lipids (glycogen) stored in complex particles—lipoproteins in the liver. The decrease in glucose levels during starvation leads to the breakdown of glycogen stores or the synthesis of glucose as a result of gluconeogenesis [69]. Epidemiological studies have indicated that diets rich in simple sugars increase both DNL and the growth of peripheral, subcutaneous, and internal adipose tissue [70]. Below, several factors, including pancreatic hormones, diet, and the body’s energy balance, are discussed in the context of their roles in the conversion of glucose into lipids and lipids into glucose. 

## 2. Hormonal and Neuronal Regulation of SUGAR Intake

The homeostasis of blood glucose or fructose levels requires strong interaction of not only the endocrine system (pancreatic hormones, stress hormones) but also communication of the nervous system.

### 2.1. Pancreatic Hormones Control Blood Glucose Homeostasis

The glucoregulation process is responsible for maintaining a constant level of glucose in the body [71]. Hormones released from pancreatic cells play an important role in this negative feedback regulation, and their release is controlled by the amount of glucose present [72]. When blood glucose levels drop, leading to hypoglycaemia, pancreatic alpha cells are activated, and glucagon is released. Glucagon is transported with the blood, e.g., to the liver, where it activates glucagon receptors, enhances the process of glycogenolysis and inhibits glycogenesis. Glycogenolysis breaks down spare glycogen into glucose and releases it into the bloodstream. During hyperglycaemia (i.e., an increase in glucose levels in the blood), insulin, another pancreatic hormone, is released. Similar to glucagon, insulin is transported with the blood to the liver but enhances the process of glycogenesis and accelerates the uptake of glucose from the blood by muscle and adipose tissue cells [73,74,75]. 

### 2.2. Sweetness Perception and Regulation

The processing of sweet taste information is related to the presence of taste receptor cells (TRCs) expressing G-protein coupled receptors (GPCRs) concentrated in the taste buds of the tongue [76]. The taste 1 receptor family and the taste 2 receptor family of type 2 TRC receptor cells are capable of detecting sweet tastes. Upon activation with a sweet molecule ligand, the GPCR receptor activates the chemosensing signalling pathway [77]. Finally, ATP is released by the semichannels, activates the purinergic receptors present on the afferent fibres of the cranial nerves of the taste buds, and sends signals to the brain taste perception areas located in the gustatory cortex [78]. In the intestines, sweet taste receptors are concentrated mainly on enteroendocrine cells that secrete bioactive molecules, e.g., hormones. Sweet taste receptors in the gastrointestinal tract are responsible for nutrient detection, glucose homeostasis, and the secretion of gastrointestinal peptides [79]. The taste information from the intestines via the vagus nerve moves to the rostral division of the nucleus of the tractus solitarius (in rodents also to the parabrachial nucleus) and then to the parvicellular part of the ventral posteromedial nucleus of the thalamus, via the ventral path to the amygdala and to lateral areas of the hypothalamus [80,81].

### 2.3. Energy-Balanced Peptide Hormones

Sugar intake and metabolism are controlled by peptide hormones, such as leptin and ghrelin. Leptin is produced by adipocytes, whereas ghrelin is secreted by gastric enteroendocrine cells [82,83]. Leptin and ghrelin, together with blood, penetrate the BBB and regulate the activity of the hypothalamic melanocortin system in the area of the arcuate nucleus (ARC) [84]. One group of ARC neurons—anorexigenic—expresses the precursor peptide pro-opiomelanocortin (POMC) and CART (cocaine and amphetamine-regulated transcript). POMC is converted into α-melanocyte-stimulating hormone (α-MSH) and serves as a melanocortin 4 receptor (MC4R) agonist. Activation of POMC/CART neurons by leptin causes anorexic effects, including reduced food intake, weight loss, the release of α-MSH, and decreased release of NPY. The second group of neurons–orexigenic–expresses neuropeptide Y (NPY) and the MC4R antagonist agouti-associated protein (AgRP). Activation of NPY/AGRP neurons by ghrelin causes increased food consumption, weight gain, AgRP release, and decreased α-MSH release [85,86]. Leptin induces weight loss by suppressing food intake, whereas ghrelin acts as an appetite stimulant signal. An HSD (rich in carbohydrates) causes an increase in leptin concentrations and a decrease in circulating ghrelin levels, which are greater than with a high-fat diet (HFD) [87].

### 2.4. Stress-Induced Bingeing “Comfort Food”

As discussed above, exposure to several stressors alters the metabolic and behavioural status of the body [88]. Stress factors activate feedback interactions along the hypothalamic–pituitary–adrenal (HPA) axis. The response to stress is release of the peptide corticotropin-releasing hormone (CRH) in the paraventricular nucleus of the hypothalamus. CRH is transferred through the hypothalamo-hypophyseal portal system to the anterior pituitary gland and stimulates corticotrophic cells to release adrenocorticotropic hormone (ACTH) from POMC. Via the bloodstream, ACTH travels to the adrenal glands, where it stimulates the secretion of cortisol, which is the most important stress-induced hormone [89,90]. Cortisol regulates glucose and lipid metabolism, appetite, food consumption, and weight gain [91,92]. Exposure to stress (prenatal and postnatal) can alter both the amount and the quality of calories consumed, and stress-induced changes in food consumption and energy balance can interact with the emotional state of humans [93,94]. The high reactivity of cortisol to stress can increase susceptibility in humans to eating palatable products rich in fat or sugar, called “comfort food” [95,96,97,98,99]. Similar to clinical observations, enhancement of palatable food intake has been reported in rodents during stress. Experimental animals choose “comfort food” to reduce depression and/or anxiety status [97,100]. As demonstrated in the limited sucrose intake paradigm, sucrose solution reduced stress responses [101,102,103]. Consumption of sucrose or highly sweet foods can limit activation of the stress system by an effect on the reward circuits in the brain [104,105]. Conversely, enhanced sugar intake can promote stress-driven emotional and addictive behaviours [50,106]. 

### 2.5. Changes in the Reward Brain System Following HSD 

The reward brain system (the mesocorticolimbic pathway) is based on brain structures and neural pathways related to rewards, motivation, and the desire for pleasure [107,108]. The reward system contains numerous interrelated structures, such as the ventral tegmental area, nucleus accumbens (NAc), prefrontal cortex (PFC), hippocampus, and amygdala [109,110]. In humans, excessive consumption of glucose or fructose predisposed individuals to changes in activity in areas of the brain known to be associated with reward, learning reward, and eating behaviour. The fructose effect was limited to the activity of neurons, whereas glucose had a wide impact on the brain [111,112,113]. A diet high in sugar, including glucose, may cause excessive distribution of glucose to the brain [114], contributing to behavioural changes in food intake [115]. 

Recent preclinical data indicate that excessive sugar intake changed the reward circuitry at neurochemical and cellular levels as well as developed addictive-like behaviours and emotional states [116,117,118,119,120,121,122]. The changes depended on sugar dietary patterns. Thus, at the neurochemical level intermittent access to sucrose enhanced the dopaminergic, opioid and cholinergic neurotransmission in the mesocorticolimbic system [123,124]. Binge-like sugar consumption facilitated dopamine (DA) release in the NAc, similarly to drugs of abuse [125]. Long-term consumption of sucrose altered nicotinic acetylcholine receptor (nAChR) expression in the NAc, whereas nAChR compounds evoked different effects on sucrose intake depending on its long-term vs. short-term exposure [126,127]. Sucrose solution given chronically (7 or 21 days) to male rats increased the level of accumbal DA neurotransmission [124,125,128]. Additionally, a 25% glucose solution over 31 days increased the expression of accumbal D1 and μ-opioid receptors in female rats [129]. Finally, a maternal HSD changed the MC4R expression in brain reward structures in male and female offspring [130,131,132].

Moreover, at the cellular levels a binge-like sucrose intake by rats for long-term (12 weeks) period, but not for short-term (4 weeks) period, resulted in alterations within the medium spiny neurons in the NAc shell (but not core) with a significant reduction in dendritic length and increased distal dendritic spine density [133]. Sucrose served as a potent modulator of neuron morphology following prolonged heavy use, as its consumption enhanced excitatory synaptic strength onto NAc DA neurons [127]. In addition to sucrose, previous studies have demonstrated similarities following acute exposure to another non-caloric sweetener saccharin at the level of the NAc [134,135,136]. Very recent study indicated that chronic (12 weeks) 5% sucrose consumption to mice resulted in a reduction in the serotonin (5-HT) receptors innervation within the PFC and dentate gyrus of the hippocampus, a reduction in the number of microglia in the latter brain structure as well as a decrease in the density of the vesicular glutamate transporter (VGLUT3) and of 5-HT/VGUT3 varicosities but not in the number of oligodendrocyte progenitor cells [137]. 

### 2.6. Behavioural Consequences of HSD 

In humans, HSD let to memory impairment and cognitive deficits [49,138], whereas it does not increase the improvement of semantic memory [139]. An observational study in children indicated that sucrose consumption was associated with the incidence of impulsivity and attention deficit hyperactivity disorder (ADHD) among 6-year-old boys and that persistent, high consumption or an increase in sugar consumption between 6 and 11 years of age was not associated with a higher prevalence of ADHD between 6 and 11 years of age [140]. 

Preclinical research reported that a higher (25%) but not low (5%) sucrose concentration given for a long time (12 weeks) to adolescent mice resulted in enhancement of hyperlocomotor response to novelty and defects in episodic and spatial memory in adulthood without modulation of learning processes [141]. In rodents, an HSD had a negative impact on aspects of behavioural tasks involving decision making and behaviour selection. A 2 h intake of sucrose over a 24-day period induced a significant spatial memory deficit as measured by site recognition in rats. Moreover, rats entering the delay discounting task had behavioural evidence of hippocampal dysfunction [142]. Hippocampus-dependent alteration in behaviours was accompanied with altered hippocampal neurogenesis such as a reduction in the proliferation and differentiation of newborn neurons in the dentate gyrus [141], which raises a positive correlation between high- and long-term exposure to sucrose during adolescent and neurocognitive defects in adulthood. Furthermore, anxiety-like or depressive-like symptoms being observed simultaneously following long-term 8–25% sucrose consumption [123,143,144,145,146,147,148,149], but not with 5% sucrose consumption [137]. In addition, a high sucrose intake (30% wt/vol) in pregnant mice induced behavioural phenotypes similar to ADHD in the offspring, manifested by increased motor activity, and impulsivity. As well as, high fructose consumption (30% wt/vol) induced hyperactive behaviour, similar to the results obtained with sucrose treatment [150]. These findings suggest that the maternal HSD (sucrose or fructose) consumption could be considered a potentially new risk factor in neurobehavioural dysregulation, such as development of ADHD and/or possibly addiction-like behaviours (see below).

## 3. Maternal HSD Overabundance as Offspring Disease Factor

Increased consumption of added sugars is visible in developing societies, especially among children and adolescents. In pregnant women limited sugar intake is recommended. The period of pregnancy and lactation is a special time for the foetus. Consistent with the developmental origins of health and disease hypothesis, exposure to external environmental factors in early life influences the proper growth and development of every organism and could play a crucial role in determining the risk of developing diseases in adulthood [151,152,153]. One important external factor is the composition and type of the maternal diet consumed during pregnancy and lactation [154]. Recently, animal studies and clinical trials have confirmed the involvement of maternal nutrition in the early life of the offspring. Improper nutrition could increase the susceptibility of offspring to diseases in early life or in adulthood [155,156]. 

Clinical observations have indicated that sugar-rich foods are desirable during pregnancy and were overeaten during the study period, suggesting that many reproductive women are exposed to the adverse effects of an HSD [118]. Carbohydrates in the maternal diet during pregnancy should make up 45–64% of daily calories, including approximately 6–9 servings of whole grains per day. Breastfeeding women require approximately 500 additional kcal/day in addition to what is recommended for nonpregnant women [157]. A serious risk to the health of the mother and the foetus is incurred by being overweight and obese. Among women of reproductive age (20–39 years old), 31.8% were obese in 2011–2012. Women with a higher body mass index before pregnancy have a greater risk of adverse perinatal outcomes [158]. HSDs during pregnancy and lactation leading to overweight and obesity can increase the risk of gestational diabetes mellitus, high blood pressure, and abnormal growth of the foetus [159,160]. Furthermore, human observational studies have suggested that lactation influences insulin and glucose homeostasis [161]. In follow-up studies, lactating diabetic women showed improved glucose tolerance and lowered fasting glucose [162]. In obese women during lactation, insulin levels and the insulin-to-glucose ratio were significantly lower, and carbohydrate utilization and total energy expenditure were higher [163]. It is also of interest that maternal diseases, promoted by HSDs in pregnancy and lactation, can be transferred to offspring with the manifestation of metabolic or neurological diseases [164,165,166,167,168,169].

In preclinical studies, HSDs during pregnancy and lactation caused effects similar to those observed in clinical trials. Apart from metabolic syndrome (weight gain and increased levels of triglycerides, cholesterol, leptin, glucose, and insulin), HSDs induced cardiac diseases and increased blood pressure in rodents [170,171]. In other studies, a maternal diet rich in fats and/or sugars during pregnancy and lactation predisposed the offspring to developing the metabolic disorders observed in the mother or promoted neurobehavioural disorders [172,173,174,175,176,177,178,179,180,181]. Maternal HSD (64% kcal) affect the memory processes, such as impaired recognition memory and spatial memory, and also disrupting the glutamate NMDA receptor in the PFC of rat offspring [182]. Moreover, “junk food” rich in sugar, eaten during pregnancy and lactation, predisposes individuals to hyperphagia due to enhancement of the choice of palatable food, also in offspring [183].

## 4. HSD as a Risk Factor to Develop SUD and Food Addiction

SUD is a serious complex disorder that affects the brain and behaviour and leads to uncontrolled use of a legal or illegal substance despite harmful consequences and unsolved issues from the perspective of public health [184]. Many factors influence SUD development contain social-age and economic situation, biological-genetic predisposition or parental drug history, and psychological-high impulsivity or sensation-seeking, determinants. Currently, the prevention of SUD is quite costly and ineffective due to the lack of appropriate therapies, the resistance and the relapse of patients to SUD. Broader SUD education and effective psychotherapy are required, and pharmacotherapy is currently being sought.

According to the World Drug Report, in 2018 nearly 1 in every 19 people (269 million people worldwide) had used drugs at least once in the previous year, and the number of users of any drug globally increased by approximately 28% over the 2009–2018 period. Of these people, in the past year, approximately 35 million had drug use disorder symptoms, corresponding to 0.7% of the population aged 15–64 years old [185]. Several drugs can involve addictive behaviour in both humans and animals. These drugs consist of psychostimulants, opioids, cannabinoids, nicotine, and alcohol. Their behavioural outcomes are realized through distinct effector mechanisms, such as neurotransmitter transporters, ion channels, and receptor proteins. As a consequence of long-term intake of all drugs of abuse—after initial positive emotions—they generate compulsive and uncontrollable motivations to seek and reuse them despite negative consequences, e.g., psychic, somatic, and/or vegetative disturbances) [186]. It is well documented that drugs of abuse—despite different pharmacological mechanisms of action via neurotransmitter transporters, ion channels or receptor proteins—share the common feature that they trigger addictive potential. The psychostimulant—cocaine rapidly increases DA neurotransmission within the mesocorticolimbic circuitry of the brain from the ventral tegmental area to the ventral striatum (=NAc) and the PFC, and induces synaptic plasticity in DA neurons after acute, passive administration and in cocaine self-administered rats [187,188]. Notably, addiction-like behaviour has also been associated with disrupted homoeostasis of other monoamine or amino acid neurocircuitries. 

It should also be indicated that the consumption of sugar or HSD can be addictive [189,190] and can predispose individuals to the risk of food addiction. At the neurochemical level, in both animals and humans there are significant similarities and overlaps between drugs of abuse and sugar, from brain neurochemistry to behaviour [189]. Long-term sugar consumption produced cocaine-like effects, changes in striatal D1 and D2 receptors, and changes in mood, possibly through its ability to induce reward and pleasure [105,119,120,122,143]. Additionally, the availability of DA precursors may be altered by prolonged consumption of HSD [191]. Sugar consumption produces cocaine-like effects, changes in striatal D1 and D2 receptors, and changes in mood, possibly through its ability to induce reward and pleasure, leading to sugar-seeking behaviour [119,120,121,123]. Sugar acts as an addictive molecule, causing overeating and withdrawal; it also affects mood, behaviour, learning, and memory [192]. These features are also characteristic for drugs of abuse [193]. More importantly, food addiction, such as SUD, is characterized by seeking and compulsive behaviours. Thus, bingeing sweet food or long-term exposure of HSD to rodents let to overweight or obesity [194], whereas sucrose or sugar withdrawal predispose them to behaviour similar to depression and anxiety [120,143]. Fructose (8% solution) given for 21 days to male rats increased bingeing behaviour following a long-term intermittent access model and decreased NAc shell neuron activation [195]. 

Moreover, recent data have indicated that diet patterns, especially maternal HFD, can increase addiction-like behaviours in offspring [130,196,197,198], but there are few sets of data on the impact of an HSD in the prenatal and postnatal periods [50,131]. As shown, a maternal HSD during pregnancy and lactation (Table 3) or postnatal HSD consumption (Table 4) impacts sensitivity to addiction-like behaviours. For example, our studies indicated that maternal HSD increased cocaine-seeking behaviour in offspring in a rat model of cocaine self-administration [130,131]. Maternal sucrose and HFCS intake increased alcohol intake in females and caused hyperactivity in response to amphetamine in male offspring [199]. Similar to the prenatal period, postnatal high-sugar intake promoted locomotor hyperactivity to cocaine [200] and amphetamine [201,202,203], whereas sugar either increased or decreased cocaine [204] and amphetamine [203,205] behaviour in the conditioned place preference. Furthermore, palatable food in amphetamine administration provoked memory impairment [206] and food intake dysregulation in rats [207] and baboons [208]. In addition, a diet rich in corn starch enhanced depressive-like behaviour in male rats after cocaine treatment [209]. The available laboratory data strongly suggest that a maternal HSD or high-sugar postnatal bingeing predisposes to changes in the sensitivity to addictive drugs; however, the behavioural outcomes depend on diet time-frame exposure and drug-dependent measurements. 

## 5. HSD Evokes Behavioural Predictors of Drug of Abuse 

Several factors have been proposed to be potentially involved in the onset and development of SUD. SUD—development and abuse—is correlated with physical and mental illnesses such as anxiety, depression, personality disorders, eating disorders, and abnormal mood changes. Among them, mood disturbances are important predictors of frequent use of drugs of abuse and problems. Anxiety disorders are a group of mental disorders characterized by a significant feeling of anxiety or fear [210]. Women are 1.5 to 2 times more likely than men to receive a diagnosis of an anxiety disorder. The significant mediators of anxiety in the central nervous system are norepinephrine, 5-HT, DA, and gamma-aminobutyric acid [211]. In fact, anxiety (especially social anxiety disorder (=social phobia)), characterized by sentiments of fear and subjectively unpleasant feelings in the face of anticipated events, was found to be a trigger for drug use in adolescence or young adulthood [212,213,214,215,216,217]. Higher levels of anxiety symptoms were associated with earlier alcohol consumption by humans [218,219] and in a rodent model of depression [220]. Moreover, anxiety triggers a greater desire for cocaine [221,222] or nicotine [223,224,225] addiction, as observed in human studies.

Similarly, depressive symptoms contribute to the use of addictive drugs. According to the WHO, 280 million people worldwide have depression, and an estimated 3.8% of the population affected, including 5.0% among adults, suffers from depression. Depression affects an estimated 15 adults (6.7%) in any given year. Women are more likely to be affected by depression than men. Among depressive symptoms, the strongest predictors of depression are crying, pessimism, changes in appetite and loss of interest, and they enhance the frequency of cocaine use or greater severity of cocaine use in current cocaine users [226,227,228,229,230]. Clinical findings have also revealed that anhedonia (=lack of interest or pleasure in activities) was associated with lifetime cocaine use and dependence [231] and enhanced the addictive effects of cocaine, leading to greater use and poorer treatment outcomes [232]. Another important predictor involved in the onset and development of SUD is impulsivity [233], which refers to a tendency to act without careful thinking or to react prematurely [234,235]. This feature is a marker of psychiatric symptoms in general and in SUD. As shown in many clinical and preclinical studies, impulsivity is a vulnerability trait for SUD and a property of SUD [236,237,238]. Moreover, variability in impulsivity could increase the odds of both heavy drinking and alcohol-related problems [239], and a significant relationship between impaired response inhibition or impulsivity and high-risk alcohol use in nonclinical populations was demonstrated [240]. Bozkurt and colleagues [241] found that—among others—self-reported trait impulsivity predicted alcohol use severity in treatment-seeking patients [242]. There have also been epidemiological studies showing a direct association between the sensation/novelty-seeking trait and SUD, especially cocaine use disorder (CUD) [243,244,245,246]. Sensation/novelty seekers are more prone to experimenting with addictive drugs [247,248]. The laboratory data support the clinical observations since sensation/novelty-seeking has been implicated in vulnerability to CUD in rodent models [249]. Thus, the sensation/novelty-seeking trait (recognized as a high-responder (HR)) trait in novelty-induced locomotor activity or high-novelty-preference trait (HNP) in novelty-induced place preference (=the propensity to choose a new environment in a free choice procedure) in rats is associated with differential sensitivity to psychostimulant drugs of abuse [250,251]. As demonstrated by Belin and colleagues, HR rats represent a “drug use prone” phenotype that causes an individual to develop CUD (but not compulsive cocaine self-administration), whereas HNP rats copy with an “addiction prone” phenotype that enhances the shift from sustained to compulsive drug intake and addiction [249].

HSDs have also been linked with emotional or mood disturbances, such as stress, anxiety, and depression. Stress—a normal, useful reaction to trigger motivation, adaptation, and reaction to the surrounding environment—at high levels can result in biological, psychological, and social problems and even serious harm to people and animals [252]. As shown, nine weeks of exposure to 15% fructose solution resulted in passive stress-coping behaviour in female mice, whereas 15–30% fructose evoked lower self-care behaviour and short-term impairment of spatial memory [253]. Interestingly, the induction of chronic social stress led to a decrease in glucose uptake, particularly in the brain tissue, which could contribute to a reduction in the incentive to reward sweet tastes [254]. The inductors of anxiety symptoms seem to be—among others—simple sugars, refined carbohydrates, and sweeteners consumed in large amounts. Limited animal studies have demonstrated an increase in anxiety-like symptoms after a chronic 7-week diet rich in sweeteners [255]. In a human study, in turn, an improvement was shown in anxiety symptoms among obese participants following a low-carbohydrate diet [256].

Depression (major depressive disorder) is a mental disorder that negatively affects feeling and thinking [257]. It is caused by a combination of genetic, biological, environmental, and psychological factors [258]. Adult female rats fed a high fructose diet (55% kcal) showed increased depressive-like behaviours in the forced swim test [259], whereas preadolescent, but not adult, male rats showed increased activity in the open field test and reduced time spent in the open arms on the elevated plus maze test. Moreover, fructose-fed rats spent more time immobile in the forced swim test [145]. Human research has revealed that high consumption of foods rich in added sugars and sweetened drinks increased the risk of depression over the next few years [260] and resulted in a high incidence and recurrence of mood disorders [261]. More recent studies have confirmed that excessive consumption of sugar-sweetened soft drinks was associated with an increased risk of depression in adults and Asian adolescents [262]. In overweight people, increased consumption of SSBs was associated with an increased incidence of a diagnosis of depression [263]. The results of studies on repetitive body-centred behaviour found that high-sugar consumption was associated with greater impulsivity without planning, whereas diets high in both saturated fat and sugar were associated with greater motor impulsiveness among study participants [264]. Large amounts of added sugar in food induced greater sensitivity to delays, greater preference for larger size, and increased size sensitivity [265], according to an earlier study in rats in the impulsive choice task [266]. 

Correlation between foetal programming and metabolic health of the offspring in human epidemiological studies of maternal HFD and HSD is strong, especially in long-term offspring implications such as obesity and diabetes. However, no data from clinical trials exist linking maternal consumption of HSD during pregnancy or postnatal consumption and predisposition to SUD. Preclinical studies emphasize the role of HSD in behavioural, neural, and neurochemical changes causing a higher vulnerability to addiction-like behaviours leading to an increased preference to consume palatable foods and sensitivity to drugs of abuse, but many more clinical trials are necessary to understand this important mechanism.

## 6. Conclusions and Perspective 

This paper has emphasized the latest data showing that HSD, added sugar, and sugar components are some predictive factors in changing behaviour. Epidemiological studies have shown that sugar intake is too high in industrialized countries. An increasing number of adults and children consume added sugar, SSBs, HFCS, and refined sugar. Preclinical studies have pointed out that a maternal HSD during pregnancy and lactation is strongly correlated with offspring’s metabolic and mental disorder development (mood disorders and compulsive or impulsive dysfunction). HSD and sugar can be addictive and can predispose an individual to the risk of food addiction. Recent limited animal studies have demonstrated that perinatal or postnatal chronic exposure to HSD or sugar (glucose and/or fructose solutions) changed the sensitivity to drugs of abuse in addiction-like behaviour models. In addition to increasing the locomotor activity of the animals, sugar in combination with the drug enhanced drug preferences and sensitivity. Then, HSD promoted an increase in depressive-like behaviour in rodents with a history of addiction-like behaviours. Finally, in vivo functional studies have confirmed that dietary sugar and drugs of abuse use the same brain structures, being a part of the reward system pathways. 

Clinical observations support preclinical findings and suggest a link between diet patterns and the risk of mental disorders. Epidemiological data showed increases in depressive and anxiety behaviours in patients with HSD histories and drug users. Sugar overconsumption can also affect mood, causing compulsivity and impulsivity repetitive behaviours, which are SUD predictors. The current clinical nonrandomized data require better exploration and research, especially since SUD is an incurable disease and a social and economic community problem, and it affects an increasing number of people of all ages, regardless of gender.

In summary, in this paper, we discussed the current knowledge on maternal and postnatal sugar intake as predictor of the development of mental disorders, including SUD. Based on the data, it is strongly recommended limitation in sugar consumption in an individual’s everyday diet, especially for children and pregnant women. Future epidemiological analyses and clinical research are needed to better explain the effect of sugar intake on the SUD development.

## Figures and Tables

**Table 1:** Classification of dietary carbohydrates. Saccharides are classified according to degree of polymerization (DP) by indication of the length of the carbohydrate chain. The sugar term includes the sum of all the mono- and di-saccharides (DP1, and DP2). Higher DPs classified as carbohydrates, such as oligo- and polysaccharides. Functional and structure divisions include subgroups with major dietary components and main natural sources.

Types (DP)	Subgroup	Major Components	Natural Sources
Sugars (DP1, and DP2)	monosaccharides	glucose, galactose, fructose	fruit, vegetables, honey, seeds
	disaccharides	sucrose, maltose, lactose	table sugar (sugar cane or sugar beet), grains, dairy products
Oligosaccharides (DP3–9)	maltooligosaccharides	maltodextrins	corn starch
Polysaccharides (DP ≥ 10)	starch	amylose, amylopectin	cereals, vegetables, processed flour products
	non-starch	cellulose, hemicellulose, pectins, glycogen	vegetables, fruits, seeds, meat

**Table 2:** Major sodium–glucose linked transporters (SGLTs) and facilitated diffusion glucose transporters (GLUTs) in several mammals’ organs expression. Transfer for glucose and galactose include possibly the same transporters. Fructose requires other transport proteins (pointed by *).

Localization	SGLTs	GLUTs
Intestine	SGLT1, SGLT3, SGLT4 *, SGLT6	GLUT2 *, GLUT5 *, GLUT7 *, GLUT12
Kidney	SGLT1, SGLT2 *, SGLT3, SGLT4 *, SGLT5 *, SGLT6	GLUT1, GLUT2 *, GLUT3, GLUT5 *, GLUT9, GLUT10, GLUT11 *, GLUT13
Liver	SGLT2 *, SGLT3, SGLT4 *	GLUT1, GLUT2 *, GLUT3, GLUT5 *, GLUT7, GLUT8 *, GLUT9
Brain	SGLT1, SGLT2, SGLT3, SGLT4 *, SGLT6	GLUT1, GLUT2 *, GLUT3, GLUT4, GLUT5 *, GLUT6, GLUT8 *, GLUT13

**Table 3:** Preclinical studies on the prenatal HSD consumption on behavioural and molecular or neurochemical consequences after drugs of abuse in offspring.

Prenatal Exposure		Animals	Drug of Abuse	Consequences Enhanced by HSD		Ref
HSD Model	Period			Behavioural	Molecular/Neurochemical	
70% carbohydrates (44% sucrose)	Pregnancy and lactation	Female WR offspring	Cocaine hydrochloride	↑ CUE- and cocaine reinstatement relapse in cocaine SA ↑ cocaine-seeking behaviour	↑ MC4R expression in the NAc, and dorsal striatum	[130]
		Male WR offspring			↓ MC4R expression in the PFC, NAc, and dorsal striatum ↑ MC4R expression in the amygdala	[131]
10% sucrose or 16% HFCS solution		Male and female SD offspring	Amphetamine	↑ female alcohol intake during alcohol training ↑ male hyperactivity in LAM	-	[199]

HSD: High-Sugar Diet; HFCS: high-fructose corn syrup; WR: Wistar rat strain; SD: Sprague Dawley rat strain; CUE: tone + light—conditional stimulus; SA: self-administration; LAM: locomotor activity measurement; NAc: nucleus accumbens; PFC: prefrontal cortex; MC4R: melanocortin 4 receptor; ↓: decrease; ↑: increase; -: no data.

**Table 4:** Preclinical studies on the postnatal HSD consumption on behavioural and molecular or neurochemical consequences after drugs of abuse.

Postnatal Exposure		Animals	Drug of Abuse	Consequences Enhanced by HSD		Ref
HSD Model	Period (Days)			Behavioural	Molecular/Neurochemical	
10% wt/vol sucrose	84	Male and female C57BL/6J mice	Cocaine	↑ female postcocaine tests locomotor activity, ↑ female sensitization to cocaine in LAM	-	[200]
0.2% saccharin (0.3 mL/trial)	15	Male WR		↓ sensitization to cocaine in discrete-trials choice procedures	-	[192]
70% from corn starch	38	Male WR		↓ immobility, head shake, and locomotion after drug treatment before FST ↓ immobility, climbing, swim, dive, and locomotion in cocaine withdrawal FST	-	[209]
8% fructose or glucose or sucrose	9	Male SD		↑ cocaine-CPP after sucrose and fructose, ↓ cocaine-CPP after glucose bingeing	-	[204]
10% wt/v sucrose	21	Female SD	Amphetamine	↑ activity and sensitivity to low dose of drug in LAM	-	[202]
10% sucrose	5	Male SD		↑ activity in LAM	-	[201]
32% (w/v) sucrose solution	21	Male LE		↑ conditioning scores in CPP	-	[205]
50% kcal from sucrose	>35	Male WR		↑ locomotor activity in LAM, ↓ sensitivity to drug in CPP, and ICSS	↓ NAc DA ↑ VTA DAT mRNA ↓ extracellular DA	[203]
60% fructose	47, 88, and 144	Female SD		↑↓SA, and spatial memory testing in water maze test	-	[206]
64.3% sugar in palatable food	30	Male LE		↑ chow intake after SA	-	[207]
75% sugar in Skittles candy	28	Male baboons (Papio cynocephalus anubis)		↓ sensitivity to candy reward during pellet meal sessions	-	[208]
32% (w/v) sucrose solution	21	Male LE	Fentanyl	↑ conditioning scores ↑ time spent in the previously drug-paired side in CPP	-	[205]

HSD: High-Sugar Diet; WR: Wistar rat strain; SD: Sprague Dawley rat strain; CUE: tone + light—conditional stimulus; SA: self-administration; LAM: locomotor activity measurement; LE: Long-Evans rat strain; FST: Forced Swimming Test; CPP: Conditioned Place Preference Test; HFHS: High-Fat High-Sugar Diet; ICSS: Intracranial Self-Stimulation; NAc: nucleus accumbens; VTA: ventral tegmental area; DAT: dopamine transporter; DA: dopamine; ↓: decrease; ↑: increase; -: no data.

## Data Availability

Not applicable.
